# INdoor Home Air Level Exploration (INHALE) Study: Protocol to Monitor Indoor Pollution in British Dwellings

**DOI:** 10.3390/ijerph22111635

**Published:** 2025-10-27

**Authors:** Thiphanie P. Riveron, Rebecca L. Cordell, Anna L. Hansell

**Affiliations:** 1 School of Chemistry, University of Leicester, Leicester LE1 7RH, UK; 2 Centre for Environmental Health and Sustainability, University of Leicester, Leicester LE1 7RH, UK; 3 Leicester NIHR Health Protection Research Unit in Chemical Threats and Hazards, University of Leicester, Leicester LE1 7RH, UK; 4 NIHR Leicester Biomedical Research Centre, Leicester General Hospital, Gwendolen Road, Leicester LE5 4PW, UK

**Keywords:** indoor air quality, dwellings, indoor pollution, sampling protocol

## Abstract

Knowledge on indoor air pollution exposure is limited. Collecting high-quality measurements in home environments is challenging, owing to the complexity of sampling options, the cost and limiting disturbance to occupants. The protocol developed for the INdoor Home Air Level Exploration (INHALE) study is designed to balance these factors by sampling indoor pollution as comprehensively as possible for a single week in the living room using non-obtrusive low-moderate cost sampling devices that are issued with easy-to-follow instructions, minimising the need for researcher visits. Indoor air pollutants included in the INHALE study were selected owing to their potential impacts on human health; these include volatile organic compounds, fungal spores, fine particulate matter, nitrogen oxides and ozone. Relevant indoor factors will also be monitored, such as temperature, relative humidity and carbon dioxide, as a proxy for ventilation, while questionnaires collect relevant information on local environment, building characteristics and participant activities, culture and social and economic status. The protocol for the INHALE study is suitable for exposure, epidemiology and intervention studies. It contributes to the development of standardised indoor sampling protocols that can be used at scale.

## 1. Introduction

Historically, air quality research has predominantly focused on outdoor air pollution, with particular focus on traffic and industrial sources of pollutants. Recently, more attention has been paid to indoor air pollution [1,2,3], driven by the fact that the general population spends around 90% of their time indoors, whether in public or private buildings [4]. Long-term exposure to poor indoor air quality can be harmful to human health, especially for vulnerable groups, including the elderly, children, and people living with respiratory diseases [3].

The indoor environment contains pollutants from both outdoor and indoor sources. Outdoor pollutants enter by infiltration and/or mechanical and natural ventilation systems, or are carried inside by the occupants and pets [5]. Outdoor sources include traffic, industry, agriculture, waste management, natural disasters (e.g., wildfire) and plants [6]. Indoor sources include building materials, furniture, decorations, combustion sources, indoor plants and personal care products [7,8]. Factors such as ventilation, relative humidity and temperature can modify indoor air pollutant concentrations and composition [9,10], as can activities taking place inside, such as cooking, cleaning, printing, building renovations, and use of solvents [7,8].

**Figure 1:**
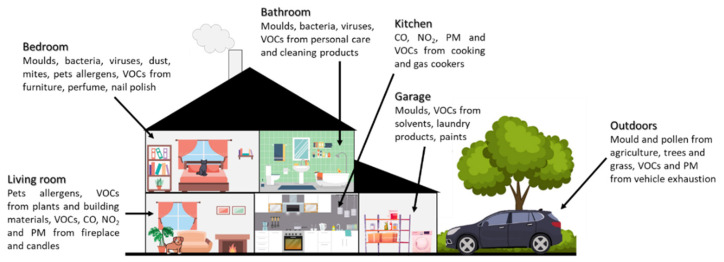
Potential indoor air pollutants and sources for each room of a dwelling. VOCs: volatile organic compounds, NO_2_: nitrogen dioxide, PM: particulate matter, CO: carbon monoxide.

Many different biological and chemical pollutants can be detected and quantified indoors, Figure 1 shows the indoor pollutants that can be found in a house depending on the activities taking place in each room. Biological pollutants, or bioaerosols, include fungal spores, bacteria, pollens, viruses and their toxins, as well as cat and dog allergens [11]. Chemical pollutants include volatile organic compounds (VOCs), particulate matter (PM) and gases, such as ozone (O_3_), carbon monoxide (CO), dioxide (CO_2_), and nitrogen dioxide (NO_2_) [8].

The 1990s saw an increase in concern and in the number of research studies into indoor air quality (IAQ) [12]. The majority of these studies were conducted in Europe, North America and East Asia [13,14,15], with a lack of studies conducted in Central Asia, the Middle East, Africa and Latin America [15]. The most studied pollutants to date have been PM, NO_2_, benzene, toluene and formaldehyde [15]. The majority of bioaerosol studies have focused on fungal species: *Alternaria*, *Aspergillus*, *Cladosporium* and *Penicillium* [13]. This makes comparisons with and between previous studies difficult, as a wide range of sampling protocols have been used, depending on the purpose of the study and the equipment available.

In the United Kingdom (UK), several reports from governmental and scientific committees [1,2,3,16] have highlighted the lack of knowledge about IAQ in British dwellings and the factors that impact it, such as building characteristics and social or demographic factors. Recent reports from the Air Quality Expert Group (AQEG) [16] and the Chief Medical Officer for England [3] concluded there was a lack of measured data, making it difficult to measure impacts on health and to consider policy interventions. Based on the government’s 2022–2023 English Housing Survey, the quality of English dwellings has generally improved over the past decades, but several potentially harmful pollutants remain present [17]. Indeed, 4% of dwellings are damp, 9% met the criteria of category 1 hazard of the Housing Health and Safety Rating System (e.g., presence of asbestos, lead, mould, heat excess) and 14% do not meet the Decent Homes Standard (e.g., reasonable state of repair, degree of thermal comfort, modern facilities and services) [17]. In addition, 3% of dwellings are considered overcrowded [17]. Based on that evidence, studies in the UK, such as those in Bradford and London [18,19], have characterised indoor pollution with different objectives and protocols.

The protocol for the INHALE (INdoor Home Air Level Exploration) study aims to achieve the following:

- Collect high-quality measurements with species-level detail for mould and VOCs, as well as more traditional air pollutants: fine particulate matter (PM_2.5_), nitrogen oxides (NO_x_), O_3_, and CO_2_, together with relative humidity, temperature and floor dust.
- Maximise acceptability (and minimise drop-out rates) by using non-obtrusive, low- or moderate-cost devices and easy-to-follow protocols.
- Be scalable to a large number of dwellings.
- Minimise staff visits to dwellings (e.g., sample collection devices can be returned by post).

The protocol draws on the published literature and has been tested for feasibility in patient and public involvement groups and in a pilot study of 70 homes.

## 2. Materials and Methods

### 2.1. Ethics

The INHALE study has been approved by the University of Leicester ethics committee (reference: 31486-tr149-ls:healthsciences). Firstly, participants are given the Participant Information Sheet (PIS), which informs them about the study and their tasks over the sampling period, with contact details provided in case participants have questions. After all the questions and concerns have been answered, and if the participant wants to join the study, they sign a consent form.

All the personal information of participants is stored securely and is accessible only to the study investigators. The paperwork is stored in a locked filing cabinet, while the digital information is stored in password-protected folders that are held on institutional servers protected by encryption. The personal information and associated ID are stored separately from the exposure data. All the data collected will be pseudonymised for data processing and analysis. Data collection, storage, usage and availability will be governed by institutional governance policies. For example, household locations will not be made public, dataset usage will be limited to the approved study researchers, and data will be held on encrypted institutional storage systems.

### 2.2. The INHALE Study Goals

**Figure 2:**
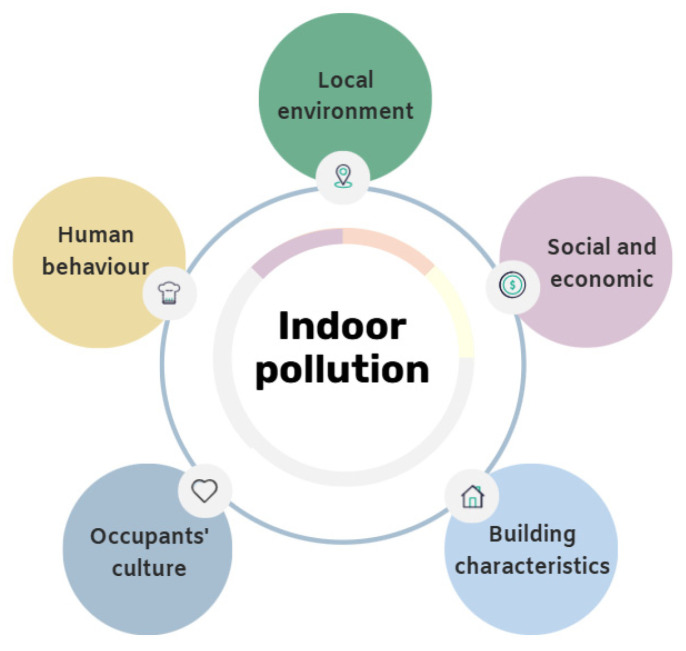
Factors that impact indoor pollution in English dwellings.

The primary goal of the INHALE study is to identify and quantify a large range of indoor pollutants to increase our knowledge on indoor pollution in British dwellings. The secondary goal is to define whether any factors (Figure 2), such as the local environment, the characteristics of the dwelling, occupant behaviour, culture or social and economic status, could impact the indoor pollution composition and concentration.

### 2.3. Recruitment

Recruitment criteria will be targeted to the dwelling and not the occupants. No inclusion or exclusion criteria will be applied. The goal will be to have a representative sample of British dwellings, including buildings of different types and ages.

To recruit, we will advertise in newsletters and social media posts of different academic organisations, associations, community centres, councils and districts. Each post or article will include the study PIS to provide more details to potential participants, as well as the contact details of the study investigators in case a potential participant has further questions. The communication and PIS will be available in English and other languages used locally to increase the recruitment and diversity of the dwellings.

### 2.4. Patient and Public Involvement and Engagement

To help develop the protocol, the investigators held patient and public involvement and engagement (PPIE) workshops. These events were an opportunity to involve communities in the project, but also to gain insight regarding the knowledge of the general public about indoor pollution, and to estimate future involvement of the communities in the study. The Indoor Air Quality report from DEFRA explained that “public awareness of the risk from chemicals is generally poor” [2].

The events were attended by individuals of different ages and cultural backgrounds, both with and without respiratory diseases. Each component of the study and protocol was tested, including measurements, questionnaires, instructions, planned communication and advertisement of the study, to ensure that the general population could perform it and that its use would not affect the well-being of future participants. The feedback was used to improve the protocol, including the creation of video instructions to complement the paper version, and the non-use of cannisters, as some participants were afraid of them.

### 2.5. Questionnaires

The Leicester Indoor Environmental Air Quality Questionnaire (Supplementary File S1) was developed to collect information about factors that potentially affect indoor air pollution (Figure 2). It contains questions about building characteristics and the outdoor environment and personal information about occupants. It was created based on previous studies undertaken in the UK [20,21], and the indoor questionnaire created by the European Community Respiratory Health Survey II [22]. For occupant ethnicity and religion, the Office of National Statistics’ ethnic group classification 20b [23] was used, as well as for religious groups [24]. To know more about human behaviour, a time–activity diary with a page per day (Supplementary File S2, example for Monday) will be given to the participants. This collects information about tasks that can impact indoor pollutant concentration and composition, as used in previous studies [19,25].

### 2.6. Instructions

The study protocol is designed for use by participants without the need for investigator visits to conduct monitoring. This reduces complexity and costs. The absence of visits can also be an advantage for people who have sensitivities about letting investigators into their homes. However, if participants do not feel confident completing the study on their own, investigators can visit their homes to set up the monitors. Written instructions provide precise details of each step that the participants will have to undertake (Supplementary Files S3–S9). Instructions have also been filmed and uploaded to a secure YouTube channel.

### 2.7. Study Design

Samples will be collected during several seasons to look at the seasonal variability in the indoor pollutants’ concentration and composition [26,27,28,29]. The influence of season on IAQ is currently unclear; several studies have reported higher concentrations in summer than in winter [27,28], while others have found the opposite [26,29].

The pollutants will be monitored only in the living room, as selected in several previous studies [27,30,31,32,33]. The bedroom was also characterised during our pilot study; however, due to privacy concerns from participants, the decision was made to only monitor pollutants in the living room. Pollutant monitors will be installed at approximately 1–1.5 m above the floor level, which is the breath height of a seated adult, as used in previous studies [27,30,34,35]. Pollutants will be monitored over a week to try to have the most representative data possible and not be too intrusive for the participants, an approach used in previous studies [28,31,36].

## 3. Results

**Figure 3:**
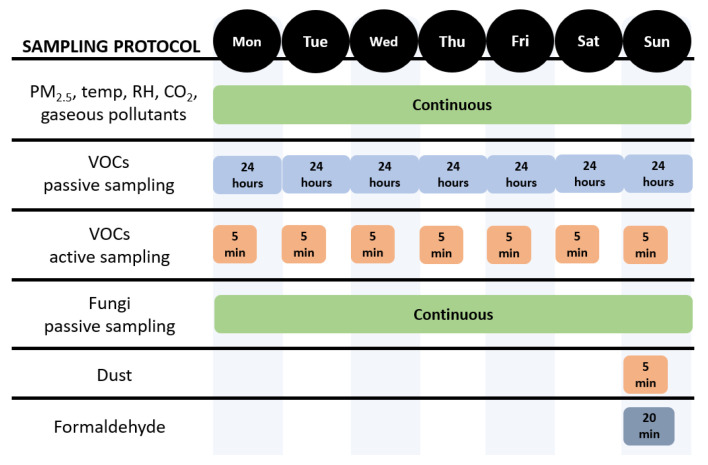
Sampling protocol developed to investigate indoor pollution in English dwellings. MON = Monday, TUE = Tuesday, WED = Wednesday, THU = Thursday, FRI = Friday, SAT = Saturday and SUN = Sunday. PM_2.5_ means particulate matter under 2.5 μm, temp means temperature, RH means relative humidity, CO_2_ means carbon dioxide, and VOCs means volatile organic compounds.

The sampling protocol focuses on indoor pollutants known for their impact on human health, especially in vulnerable populations such as children, the elderly and those with pre-existing disease. The indoor pollutants of interest are fungal spores, VOCs, PM_2.5_, gaseous pollutants (O_3_ and NO_x_), and metals (measured in dust), as well as indoor factors such as relative humidity, temperature and carbon dioxide (CO_2_) (Figure 3).

### 3.1. Volatile Organic Compounds (VOCs)

#### 3.1.1. Sampling Protocol

To identify and quantify specific VOCs in dwellings (as well as total VOCs), two different options are offered depending on the purpose of the sub-study: active (option 1) and passive (option 2) sampling methods.

- Option 1: active sampling for untargeted analysis

**Figure 4:**
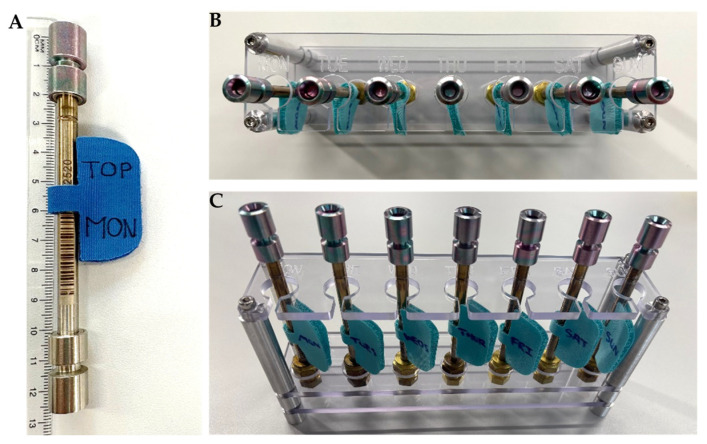
Images of the sorbent tubes used for sampling VOCs and the adaptation made to ease the protocol. (**A**) Sorbent tube for active sampling. (**B**,**C**) The rack with the sorbent tubes for passive sampling. MON = Monday.

Carbograph 1TD/Tenax TA sorbent tubes (Markes International Ltd., Bridgend, UK) will be used. A daily sample will be collected in the living room during the morning for 5 min by using a pump set-up at 200 mL·min^−1^ from Enthalpy Analytical (Exeter, UK). In total, eight labelled tubes (Figure 4A) will be given per dwelling, one per day and a field blank. Instructions for the participants are detailed in Supplementary File S3.

- Option 2: passive sampling for targeted analysis

Carbopack 5TD sorbent tubes (Markes International Ltd., Bridgend, UK) will be installed in a labelled rack (Figure 4B,C) in the living room at a height of 1–1.5 m above the floor, to obtain a 24 h mean VOC concentration. The detailed protocol is explained in Supplementary File S4. Each participant will have eight labelled tubes, one per day and a travel blank. Every 24 h, the participants will have to remove the diffusion-locking caps (Markes International Ltd., Bridgend, UK) from the corresponding tube and put on a diffusion cap (Markes International Ltd., Bridgend, UK) to allow the passive sampling.

#### 3.1.2. Samples and Data Analyses

Gas chromatography (GC) coupled with mass spectrometry will be used for passive samples. The GC will be equipped with a DB-5MS column (length × internal diameter × film thickness, 60 m × 0.25 mm × 0.25 µm) (Agilent Technologies Ltd., Stockport, UK). The GC oven will be programmed with an initial temperature of 35 °C, followed by a ramp of 2.8 °C·min^−1^, up to 130 °C, followed by a second ramp of 4 °C·min^−1^, up to 220 °C, followed by a third ramp of 25 °C·min^−1^, up to the final temperature of 320 °C for 10 min. The total run time will be 70 min. Data will be acquired in MassHunter GC-MS Acquisition B.07.04.2260 (Agilent Technologies Ltd., Stockport, UK). The data will be processed using MassHunter MS quantitative analysis (Agilent Technologies Ltd., Stockport, UK).

Two-dimensional gas chromatography coupled with mass spectrometry and a flame ionisation detector will be used for active samples, and untargeted analysis will be performed as detailed in Wilde et al. [37]. The data will be processed by using GC Image™ v2.6 along with Project and Image Investigator (JSB Ltd., Horsham, UK), as detailed in Wilde et al. [38].

### 3.2. Formaldehyde

Two methods will be used to quantify formaldehyde based on the US EPA protocol [39], by using our instrument or an external company. For the first option, samples will be taken into a cartridge coated with 2,4-dinitrophenylhydrazine and analysed using a Waters Alliance e2695 coupled to an APGC V2.0 (Waters, Milford, MA, USA). For the second option, IAQ Home Survey from Enthalpy Analytical will be used. The sample will be collected based on their recommended protocol and analysed by Waverton Analytics (Nantwich, UK).

### 3.3. Fungal Spores

#### 3.3.1. Sampling Protocol

Passive sampling will be used to quantify and identify fungal spores present in the air, based on the method developed by Shelton et al. [40]. The choice was made to minimise noise and electricity use. A 3D-printed sampler will hold the MicroAmpTM clear adhesive film (ThermoFisher Scientific, Loughborough, UK), allowing a week’s sampling in the living room, at a height of 1–1.5 m above the floor. The complete protocol is detailed in Supplementary File S5, as well as recommendations to avoid interference from people or pets to not affect the sampling. The lid will be removed for sampling, then replaced to ensure the integrity of the sample during transportation.

#### 3.3.2. Samples and Data Analyses

The samples will be transferred to a microscopic slide and dyed with lactophenol cotton blue, allowing spore identification and count with a light microscope at magnification ×400 (Zeiss, Oberkochen, Germany), as detailed by Sterling et al. [41]. Spores will be identified by genus based on various identifiable characteristics, as explained by Lacey and West [42].

### 3.4. Dust Samples

#### 3.4.1. Sampling Protocol

Dust samples will be collected by vacuuming the floor for five minutes using the protocol from Vesper et al. [43] and using the participant’s own vacuum cleaner. The instructions are explained in Supplementary File S6. Samples will be collected into DUSTREAM collector and filter (Indoor Biotechnologies, Cardiff, UK) from the living room and bedroom floor [43] to increase the likelihood of having enough dust for analyses. This method was selected owing to its capacity to collect a large amount of dust compared to wipes.

#### 3.4.2. Samples and Data Analyses

DNA sequencing will be performed on dust samples. DNA from three fungal regions (ITS1, ITS2 and the D1/D2 region of LSU) will be amplified. The resulting amplicons will be sequenced and analysed as described in Marczylo et al. [44]. Alternatively, q-PCR will be performed by selecting a short sequence of DNA unique to the fungal species of interest to create a primer that will allow us to amplify and quantify the DNA present in the sample by using a real-time PCR system, as explained by Fairs et al. [45]. For metal analysis, dust will be digested based on Dingle et al.’s protocol [46]; then, elemental analysis will be performed using Inductively Coupled Plasma Mass Spectrometry.

#### 3.4.3. Future Potential Use of the Dust Samples

Dust samples are important to collect as a large range of pollutants can be detected in this type of sample. A non-exhaustive list of pollutants includes semi-VOCs (SVOCs), such as flame retardants, which can be assessed using gas chromatography [47,48]; toxins can be derivatised and analysed by gas chromatography–mass spectrometry [49]; allergens (e.g., House Dust Mite (HDM) Dermatophagides pteronyssinus, HDM Dermatophagoides farinae, cat Felis domestica, dog Canis familiaris) can be identified using the ELISA technique [50,51,52]; and bacteria can be examined by sequencing the V4 region of 16S rRNA with the 515F/806R primers [53].

### 3.5. Fine Particulate Matter and Gaseous Pollutants

#### 3.5.1. Sampling Protocol

Two options are offered depending on the goal of the study. With the first option, a weekly mean concentration of PM_2.5_ can be obtained, and the filters can be used for further chemical characterisation. The second option allows for the real-time monitoring of PM_2.5_, O_3_ and NO_x_ to identify potential sources or events linked to an increase in those pollutant concentrations.

- Option 1: gravitometry

Weekly mean PM_2.5_ concentrations will be determined by the gravimetric technique [54], using the UPAS V2 (Access sensor technologies, Fort Collins, CO, USA) with a PM_2.5_ 2 L·min^−1^ inlet, holding a Whatman Grade QM-A Quartz Air Sampling Filter, 37 mm Circle (Cytiva, Cardiff, UK). The UPAS was selected owing to its small size and the low amount of noise it makes. The UPAS will sample non-stop over a week in the living room, at a height of 1–1.5 m above the floor. The instructions are detailed in Supplementary File S7.

- Option 2: sensor for PM_2.5_ and gaseous pollutants

Real-time continuous PM_2.5_, O_3_ and NO_x_ concentrations will be monitored using an optical particulate counter to monitor PM_2.5_ and electrochemical sensors to monitor O_3_ and NO_x_ (e.g., Zephyr, EarthSense, Leicester, UK; Space Pro, Airthings, Oslo, Norway). The concentrations of PM_2.5_, O_3_ and NO_x_ are monitored every five minutes. The instructions are detailed in Supplementary File S8. The monitor will sample non-stop over a week and will be installed in the living room, at a height of 1–1.5 m above the floor. The monitors will be sent once a year to the manufacturer for calibration.

#### 3.5.2. Data Analysis

Data processing to determine the PM_2.5_, O_3_ and NO_x_ concentrations, as well as quality control, will be conducted using RStudio version 2022.07.0 (Boston, MA, USA) with the packages tidyverse [55], stargazer [56], patchwork [57], dplyr [58], MASS [59] and ggplot 2 [60]. Quality control will be performed for each dataset by performing a range check to identify implausible values, such as negative concentrations or extreme outliers (e.g., sudden spikes or drops, missing data, and sensor flatlining) by creating time series plots and statistical thresholds, such as a z-score.

### 3.6. Temperature and Relative Humidity (RH)

#### 3.6.1. Sampling Protocol

The temperature and RH inside a home are important factors to monitor, owing to their potential impact on indoor pollutants’ composition and concentration, including fungal species. The CO_2_ smart (EviSense, Eindhoven, The Netherlands) monitor will be used to measure temperature and RH. CO_2_ concentration can be used as a ventilation proxy. The monitor will be installed in the living room, at 1–1.5 m above the floor, and samples will be taken every five minutes over a week. The full protocol is detailed in Supplementary File S9. The CO_2_ smart was selected owing to its small size and large data storage capacity.

#### 3.6.2. Data Analysis

The data will be analysed and quality control will be performed using RStudio, as explained in Section 3.5.2.

### 3.7. Data Analysis Plan

#### 3.7.1. Data Pre-Processing

VOCs will be selected based on their limit of detection (LOD), calculated according to the International Union of Pure and Applied Chemistry [61]. Experimental LODs will be defined as three standard deviations above the mean signal from field blanks collected [61], usually corresponding to one blank for each dwelling. Only VOCs meeting the frequency of observation threshold with a signal above their LOD for more than 50% of the samples will be included in the analysis.

Exposure data pre-processing will start with quality control tests, performed with RStudio version 2022.07.0 (Boston, MA, USA) with the packages tidyverse [55], stargazer [56], patchwork [57], dplyr [58], MASS [59] and ggplot 2 [60]. Quality control will be performed for each exposure dataset by performing a range check to identify implausible values, such as negative concentrations, extreme outliers (e.g., sudden spikes or drops), missing data, and sensor flatlining or missing data by creating time series plots and statistical thresholds, such as a z-score. The data will be handled appropriately for the issue (e.g., high concentrations, if an artefact is not of interest, sensor/instrument problems resulting in the need to delete data). Descriptive analysis will be performed by examining weekly and daily concentrations (mean or median).

#### 3.7.2. Statistical Analysis

The indoor pollutant concentrations will be stratified based on the identified factors influencing indoor pollution (Figure 2), such as the building type or age, the number of occupants or their ethnicity, etc. To compare the difference between two groups, the Mann–Whitney test will be performed, and for among three or more groups, the Kruskal–Wallis test will be performed [62] using GraphPad Prism version 9.0.0 for Windows (GraphPad Software, San Diego, CA, USA). Principal component analysis (PCA) will be performed to identify source profiles and relationships among the exposure data. R package “corrplot” [63], on RStudio, will be used to perform the PCA analysis and the Hopins statistics. Moran’s I spatial autocorrection will be calculated using ArcMap 10.8 ArcGIS (Esri, Redlands, CA, USA) [64]. *p*-values less than 0.05 will be considered significant.

To determine the potential health impact of being exposed by inhalation to indoor pollutants, hazard quotient (HQ) and cancer risk (CR) will be calculated. For VOCs, US Environmental Protection Agency references will be used, and for PM_2.5_, O_3_ and NO_x,_ the World Health Organisation annual exposure guideline will be used [65]. HQ and CR will be calculated based on the Centre for Disease Control and Prevention formula [66].

### 3.8. Local Environment

To examine the impact of the local environment near the dwelling (e.g., urban, suburban, woodland, arable, major roadway, etc.), satellite data from the Copernicus Sentinel-2 mission from the European Space Agency will be used [67,68]. The data will be analysed with ArcMap 10.8 ArcGIS (Esri, USA) [64]. A buffer of 1 km around the dwellings’ postcode will be selected, and a ratio for each environment present will be determined. ArcGIS will also be used to determine the distance to any major or minor roads based on the Morley and Gulliver model [69].

To determine if the dwellings are located in an urban or rural area, the lower layer super output area (LSOA) code corresponding to the dwellings’ postcode will be determined from the Census 2021 geographics by the Office of National Statistics. Then, the LSOA codes will be compared to the 2021 Rural Urban Classification tables to determine if the dwelling is in an urban or rural area.

### 3.9. Social and Economic Status

Socioeconomic status will be assessed based on the postcode of the dwelling and its corresponding deprivation indices using the English indices of deprivation 2019 from the Ministry of Housing, Comunities & Local Government [70]. The indices available include the following: Index of Multiple Deprivation Decile, Income Decile, Employment Decile, Education and Skills Decile, Health and Disability Decile, Crime Decile, Barriers to Housing and Services Decile, Living Environment Decile, Income Deprivation Affecting Children Index Decile and Income Deprivation Affecting Older People Index Decile.

### 3.10. Feedback

The participants will receive bi-annual reports on the INHALE study, which include updates on the study (e.g., number of participants recruited, percentage of data analysis) and aggregated results when available. The participants will be asked if they prefer to receive a digital or paper version of the report. At the end of the study, the participants will receive a report on the results of the study.

### 3.11. Financial Compensation

All participants will receive a voucher in recognition of their time and effort. The postage labels will be provided by the study. Two monitors require electricity and the cost of their use has been determined to be less than GBP 2, based on the price of electricity in winter 2024/25. The participants can complete a form to request financial compensation for the electricity use.

## 4. Discussion

Developing a protocol to monitor indoor pollution in homes is challenging. The protocol developed for the INHALE study aims to provide an accurate proxy of personal exposure, be minimally invasive, require minimal intervention from the participants and respect their well-being without affecting the quality of the samples collected.

Data on the composition of the home environment are limited, owing to the complexity of accessing dwellings and the costs of such a study. The UK government has had less focus to date on indoor air quality than some European governments. Germany, Finland and Greece have carried out national studies to characterise indoor air quality [71]. However, over the past few years, several reports from UK governmental and scientific committees [1,2,3,16] acknowledge the lack of data on indoor pollution in British dwellings, leading to an increase in studies on this topic led by several universities using differing protocols [18,19]. However, the number of homes and indoor environments examined has been much smaller to date than those examined in European studies, which have standardised protocols making comparison between different indoor environments possible.

As mentioned in the Royal Chemistry Society’s Indoor Air Quality report, setting up an institute such as the “Indoor Air Quality Observatory” created by the French government [72] would potentially help to improve information exchange and standardisation of measurements to provide better health studies and interventions and, therefore, evidence to support policy interventions. The UK government has already reacted since the unfortunate death in 2020 of Awaab Ishak, a two-year-old boy living in a mouldy home, by publishing guidance on damp and mouldy homes for social housing [73] and promulgating Awaab’s law to enforce local authorities to renovate mouldy homes [74]. However, information on mould prevalence in indoor air in homes (whether visible or not), mould species present and quantification of spore concentrations is currently extremely limited or not available for the general population.

The outcomes from the INHALE study can help inform whether concentrations of indoor air pollutants conform to current international and national guidelines. For example, the World Health Organisation Air Quality Guidelines for ambient air can potentially be applied to indoor air, which cover CO, NO_2_, SO_2_, PM_10_, PM_2.5_ and O_3_ [65] and the Public Health England Indoor Air Guideline covering 11 VOCs [75]. Regarding fungi, the data collected during the INHALE study could help inform the creation of regulations or guidelines on indoor mould, such as those produced by the Portuguese Government (Declaração de Retificação n.º 52/2013) [76].

The protocol of the INHALE study has been developed to be used widely, for example, for epidemiological studies to define the impact of indoor pollution on vulnerable populations, as well as for mitigation interventions, and will be used in different indoor environments (e.g., workplace, schools). Each of the sub-studies will have its own research hypothesis. Sample size calculations will be undertaken, depending on the research hypothesis. Additional confounder information, stratification criteria or particular sources of bias will be investigated depending on the research hypothesis being investigated. In addition, some adjustments may be necessary (e.g., additional sampling in the kitchen or bedroom) but should not affect the well-being of the participants. Each sub-study will ensure it has a representative population (e.g., social and economic status, ages, regions). Each of the sub-studies should conduct a pilot phase when using the INHALE protocol in different populations to identify the need for potential modification. For example, the Leicester Indoor Environmental Air Quality Questionnaire asked only about the number of pets and not their species; so far, participants always specified these (cat, dog, etc.), but this may not be the case in all situations.

To finish, we hope that the INHALE study will allow us to gain a greater understanding of indoor air pollution in British dwellings and the factors impacting it. This will aid research into the health effects of indoor air pollutants and influence the future development of existing and new guidelines. We hope that the publication of the INHALE study’s protocol will inform the creation of a standardised indoor air sampling protocol.

## Data Availability

As this study is a protocol, no data are currently available.

## Supplementary Materials

The following supporting information can be downloaded at https://www.mdpi.com/article/10.3390/ijerph22111635/s1: Supplementary File S1: Leicester indoor environmental questionnaire; Supplementary File S2: Time-activity diary; Supplementary File S3: VOCs sampling protocol; Supplementary File S4: VOCs sampling protocol; Supplementary File S5: Mould sampling protocol; Supplementary File S6: Dust sampling protocol; Supplementary File S7: Particulate sampling protocol; Supplementary File S8: Particulate sampling protocol; Supplementary File S9: Other pollutants sampling protocol.

## Abbreviations

The following abbreviations are used in this manuscript:

AQEG	Air Quality Expert Group
CO	Carbon monoxide
CO_2_	Carbon dioxide
DEFRA	Department for Environment, Food and Rural Affairs
DNA	Deoxyribonucleic acid
GC	Gas chromatography
GC-MS	Gas chromatography coupled to Mass Spectrometry
HDM	House Dust Mite
IAQ	Indoor air quality
INHALE	INdoor Home Air Levels Exploration
ITS1	Internal transcribed spacer 1
ITS2	Internal transcribed spacer 2
LSOA	Lower layer super output area
LSU	Large subunit
MS	Mass Spectrometry
NO_2_	Nitrogen Dioxide
NO_x_	Nitrogen oxides
O_3_	Ozone
PCA	Principal Component Analysis
PCR	Polymerase Chain Reaction
PIS	Participant Information Sheet
PM_2.5_	Fine particulate matter
PM_10_	Particulate matter
PPIE	Patient and Public Involvement and Engagement
q-PCR	Quantitative Polymerase Chain Reaction
RH	Relative Humidity
rRNA	Ribosomal ribonucleic acid
SVOCs	Semi-Volatile Organic Compounds
UK	United Kingdom
USA	United States of America
US EPA	United States Environmental Protection Agency
VOCs	Volatile Organic Compounds
