# Supplementary material for: INdoor Home Air Level Exploration (INHALE) Study: Protocol to Monitor Indoor Pollution in British Dwellings
Source: Int J Environ Res Public Health. 2025 Oct 27;22(11):1635. doi: 10.3390/ijerph22111635 (PMC12653005; doi:10.3390/ijerph22111635)
Supplement: Supplementary file 1 [file ijerph-22-01635-s001.zip › Supplementary Files S1.pdf]

Leicester indoor environmental air quality questionnaire

SELF-ADMINISTRATED
UNITED KINGDOM VERSION

2022
University of Leicester

This questionnaire is part of a study to evaluate the potential sources of indoor pollutants in homes. Please tick the box where applicable.

Home history

Age of the building (years)?

50+	50-40	40-30	30-20	20-10	10-1	<1
☐	☐	☐	☐	☐	☐	☐

Have you recently (in the last 2 years) changed?	**Yes**	**No**
Your bedroom flooring	☐	☐
Your living room flooring	☐	☐
Your bedroom wall coverings	☐	☐
Your living room wall coverings	☐	☐

Have you observed the presence of?		
Condensation	☐	☐
Damp	☐	☐
Visible mould	☐	☐
Smell of mould	☐	☐

Inhabitant characteristic

Number of adults living in the home:…………………………………………..

Number of children (aged under 18) living in the home:…………………………………………..

Number of pets living in the home:…………………………………………..

How long have you lived in this home (years)?

50+	50-40	40-30	30-20	20-10	10-1	<1
☐	☐	☐	☐	☐	☐	☐

What is your home ownership type?

☐ I own my home (this can include co-ownership with family/spouse etc.)
☐ I rent my home privately from a third party
☐ My home is part of a housing association
Other (specify) ………………………………………………………………………………………………………

How many hours do you spend approximately in your home per day **during the week**?

<8	8-10	10-12	12-14	14-16	16-18	18-20	>20
☐	☐	☐	☐	☐	☐	☐	☐

How many hours do you spend approximately in your home per day **during the weekend**?

<8	8-10	10-12	12-14	14-16	16-18	18-20	>20
☐	☐	☐	☐	☐	☐	☐	☐

Please tick the box that describes your ethnicity:

- ☐ Asian, Asian British or Asian Welsh: Bangladeshi
- ☐ Asian, Asian British or Asian Welsh: Chinese
- ☐ Asian, Asian British or Asian Welsh: Indian
- ☐ Asian, Asian British or Asian Welsh: Pakistani
- ☐ Asian, Asian British or Asian Welsh: Other Asian
- ☐ Black, Black British, Black Welsh, Caribbean or African: African
- ☐ Black, Black British, Black Welsh, Caribbean or African: Caribbean
- ☐ Black, Black British, Black Welsh, Caribbean or African: Other Black
- ☐ Mixed or Multiple ethnic groups: White and Asian
- ☐ Mixed or Multiple ethnic groups: White and Black African
- ☐ Mixed or Multiple ethnic groups: White and Black Caribbean
- ☐ Mixed or Multiple ethnic groups: Other Mixed or Multiple ethnic groups
- ☐ White: English, Welsh, Scottish, Northern Irish or British
- ☐ White: Irish
- ☐ White: Gypsy or Irish Traveller
- ☐ White: Roma
- ☐ White: Other White
- ☐ Other ethnic group: Arab
- ☐ Other ethnic group: Any other ethnic group:...
- ☐ Prefer not to say

Please tick the box that describes your religion:

- ☐ No religion
- ☐ Christian
- ☐ Buddhist
- ☐ Hindu
- ☐ Jewish
- ☐ Muslim
- ☐ Sikh
- ☐ Any other religion:..............................
- ☐ Prefer not to say

Home characteristic

House type:	Terraced	Detached	Semi-detached	Flat	Bungalow	Other (specify)
	☐	☐	☐	☐	☐	....

Do you have any of this equipment in the mentioned rooms?	**Living room**	**Bedroom**
Air purifier		
Humidifier		
Scented product(s) (candles, diffuser etc.)		
Rug		

What is the wall covering in the mentioned rooms?	Living room	Bedroom
Paint		
Wallpaper		
Tiles		

Other (specify)………

What is the floor covering in the mentioned rooms?	Living room	Bedroom
Carpet		
Laminate		
Tiles		
Floorboard		
Wood		

Other (specify)………

In your home, do you have?	**Yes**	**No**	**Don't know**
Cellar			
Wall cavity insulation			
Attached garage			
Double-glazing windows			
Garden			

If you have a garden, do you have?		
Tree(s)		
Running water (e.g. stream, river)		
Pond		
Compost heap		

	Presence of fuel-burning appliance? (e.g gas boiler, wood burning stove, oil heater, gas hob/cooker)		Type of fuel-burning appliance	How many days per week is it used?	How many hours per day is it used?	Has appliance been Checked by an engineer in the last 12 months?		
	Yes	No				Yes	No	Don't know
Living room /lounge	☐	☐	………………………	……….	……..	☐	☐	☐
Bedroom	☐	☐	………………………	……….	……..	☐	☐	☐
Kitchen	☐	☐	………………………	……….	……..	☐	☐	☐
Other room (please specify)	☐	☐	………………………	……….	……..	☐	☐	☐
