# Supplementary material for: INdoor Home Air Level Exploration (INHALE) Study: Protocol to Monitor Indoor Pollution in British Dwellings
Source: Int J Environ Res Public Health. 2025 Oct 27;22(11):1635. doi: 10.3390/ijerph22111635 (PMC12653005; doi:10.3390/ijerph22111635)
Supplement: Supplementary file 1 [file ijerph-22-01635-s001.zip › Supplementary Files S2.pdf]

Time activity diary

Monday

Cooked breakfast: YES / NO
if YES, what did you cook: ……………………………………………………………………
…………………………………………………………………………………………………..
if YES, did you use the extractor hood/fan: YES / NO

Cooked lunch: YES / NO
if YES, what did you cook: ……………………………………………………………………
…………………………………………………………………………………………………..
if YES, did you use the extractor hood/fan: YES / NO

Cooked dinner: YES / NO
if YES, what did you cook: ……………………………………………………………………
…………………………………………………………………………………………………..
if YES, did you use the extractor hood/fan: YES / NO

Cooked something else: YES / NO
if YES, what did you cook: ……………………………………………………………………
if YES, did you use the extractor hood/fan: YES / NO

Did you take a shower/bath: YES / NO
if YES, did you use the extractor fan: YES / NO

Did clean your home: YES / NO
if YES, which type and which product: ……………………………………………………
…………………………………………………………………………………………………..
if YES, in which room: ……………………………………………………………………...

Did you use scented product (perfume, diffuser, candle etc.): YES / NO
if YES, which type:…………………………………………………………………………
if YES, in which room: ……………………………………………………………………...

Did you open your windows / doors to ventilate you house: YES / NO
if YES, in which room: ……………………………………………………………………...

Did you use a heating sources (central heating, boiler, open fires): YES / NO
if YES, for how long: ……………………………………………………………………...

Time spent in our home (estimate): ……………………………………………………....
……………………………………………………………………………………………………

Other relevant activities (e.g. smoking, manicure, etc.): ………………………………………
……………………………………………………………………………………………………
……………………………………………………………………………………………………
……………………………………………………………………………………………………
