# Supplementary material for: INdoor Home Air Level Exploration (INHALE) Study: Protocol to Monitor Indoor Pollution in British Dwellings
Source: Int J Environ Res Public Health. 2025 Oct 27;22(11):1635. doi: 10.3390/ijerph22111635 (PMC12653005; doi:10.3390/ijerph22111635)
Supplement: Supplementary file 1 [file ijerph-22-01635-s001.zip › Supplementary Files S3.pdf]

VOCs sampling protocol

Active sampling method

You should have two boxes labelled short-term samples, containing 8 tubes, a pump and a pair of cotton gloves. Each tube is labelled for each day of the week and a blank.

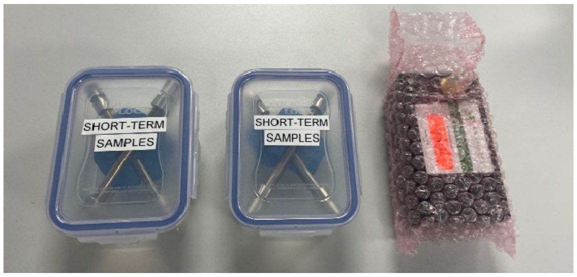

- Please remove the bubble wrap protecting the pump.

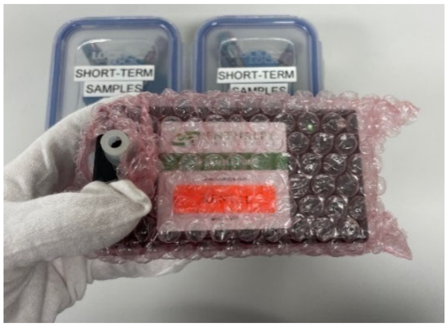

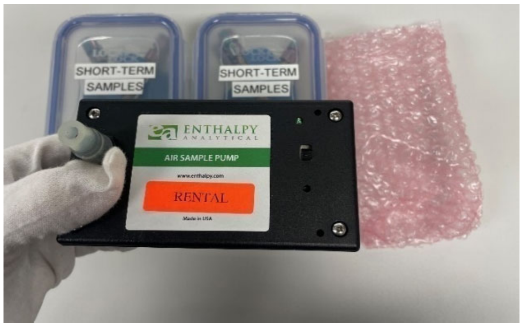

- Take the tube corresponding to the correct day. In the picture, the tube for Monday was selected.

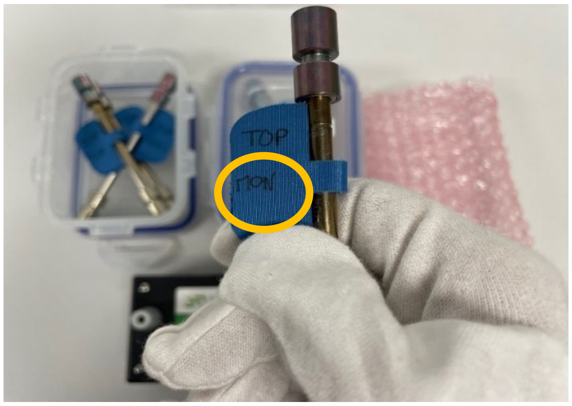

- Take off both caps, as shown in the picture below. Do not take off the label.

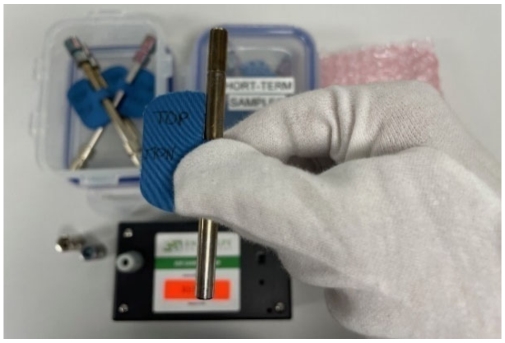

- A "TOP" sign is written on the label and indicates the top of the tube, which is shown in the picture below.

- Insert the tube into the pump, as shown in the picture below, ensuring the tube is pointing in the correct direction. The top of the label must be pointing to the air, and the bottom has to be in the pump.

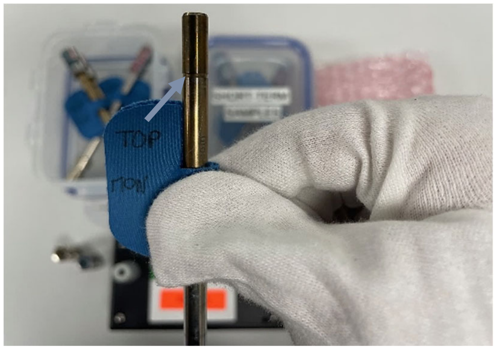

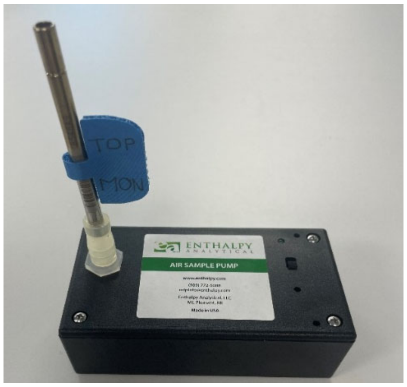

- Place the pump on a table at a height of approximately 1-1.5m.

- Turn ON the pump by pushing the switch up into the ON position.

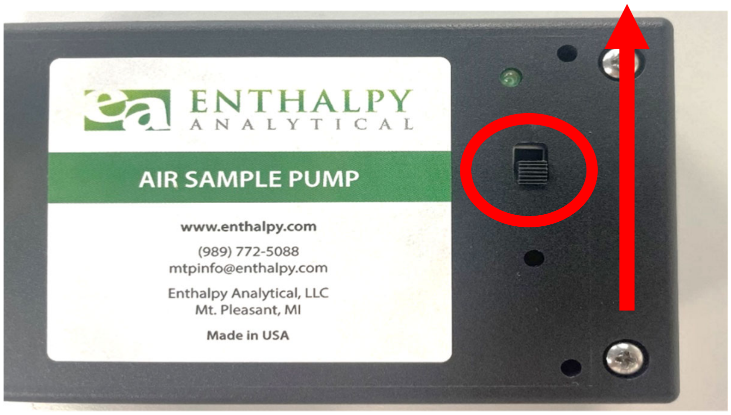

- The LED light will turn green when the pump is ON, as shown in the picture below.

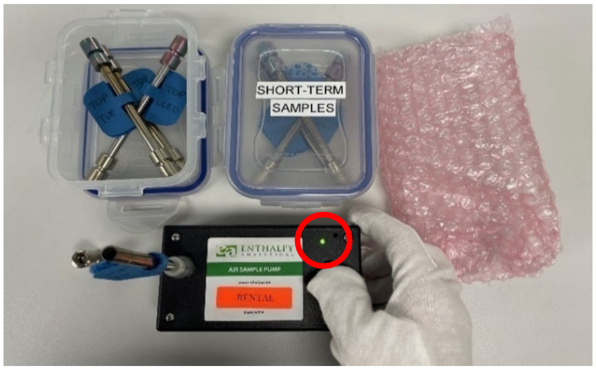

- Leave the pump ON for 5 minutes (using a timer).

- Carefully remove the sample tube from the pump tubing.

- Cap the tube and place it in the relevant box (labelled short-term samples).

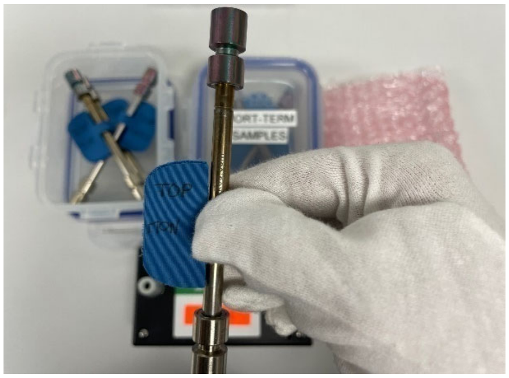

- Repeat this protocol every day.

- At the end of the week, place the tubes in the relevant box (labelled short-term samples), put the bubble wrap on the pump and put the boxes, pump and gloves in the package.

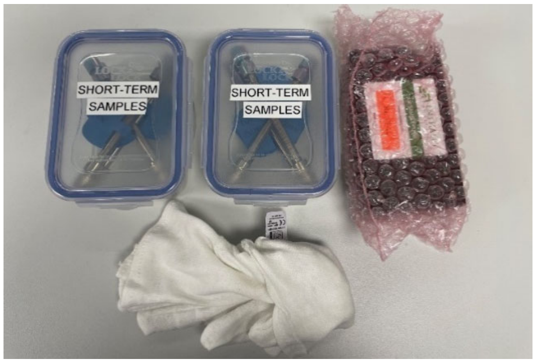

- A tube labelled “BLANK” will be present in the box; please don’t use it, as it is a control.
