# Supplementary material for: INdoor Home Air Level Exploration (INHALE) Study: Protocol to Monitor Indoor Pollution in British Dwellings
Source: Int J Environ Res Public Health. 2025 Oct 27;22(11):1635. doi: 10.3390/ijerph22111635 (PMC12653005; doi:10.3390/ijerph22111635)

VOCs sampling protocol

Long-term samples

You should have two boxes labelled long-term samples, containing 8 tubes, a rack and a pair of cotton gloves. Each tube is labelled for each day of the week, and a blank.

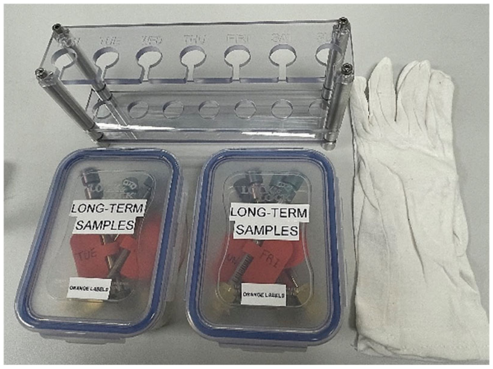

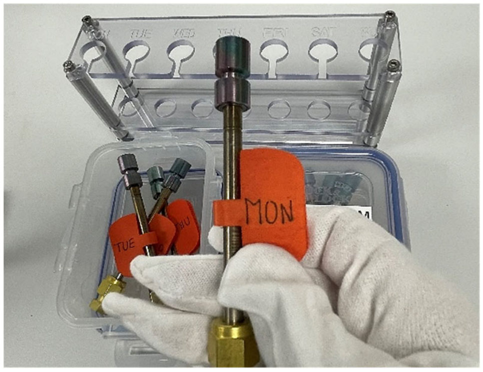

- Each hole of the rack is labelled for a day of the week. Please put the corresponding labelled tube in its hole, as per the picture, facing in the direction shown in the pictures below. The brass caps must be at the bottom of the rack, and the larger silver cap at the top. Please, leave the bank tube in the box without closing the lid.

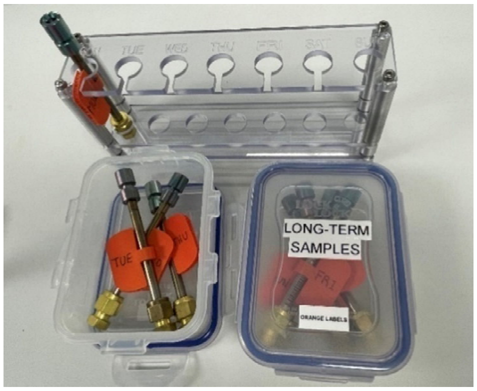

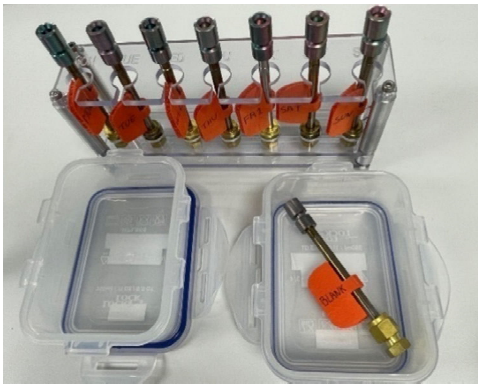

- Place the rack/tubes in your living room, at a height of approximately 1-1.5m.

- In one of the boxes, you should have the small silver mesh cap, shown in the picture below.

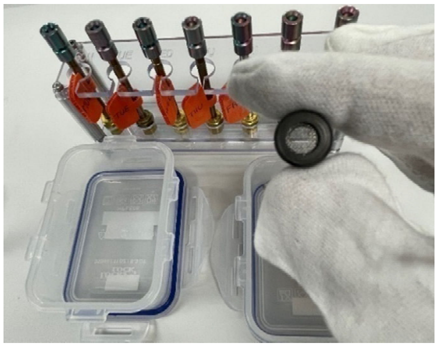

- Take off the large silver cap of the Monday tube, and put on the small mesh cap instead.

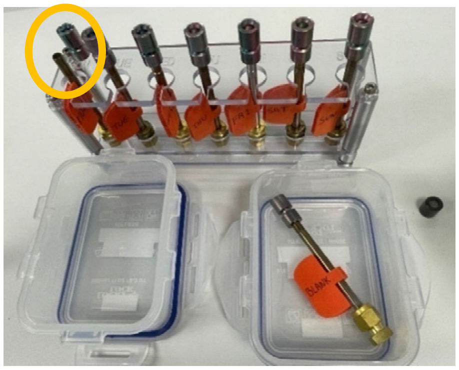

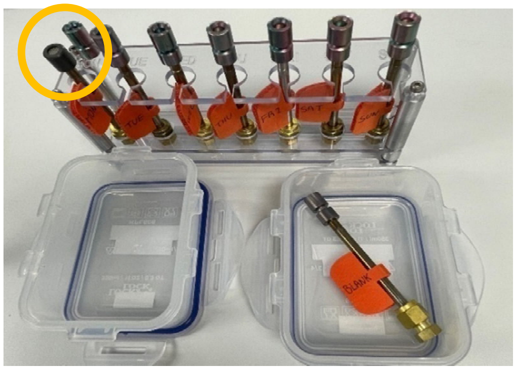

- Leave it for 24 hours.

- Every 24 hours, remove the large silver cap from the relevant tube (e.g. TUES on Tuesday, WED on Wednesday, etc) and put the small mesh cap instead, and leave it for 24 hours.

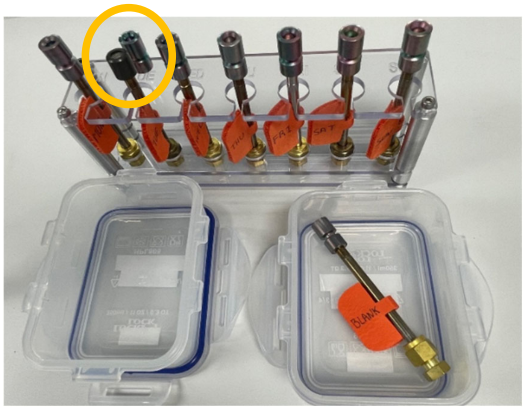

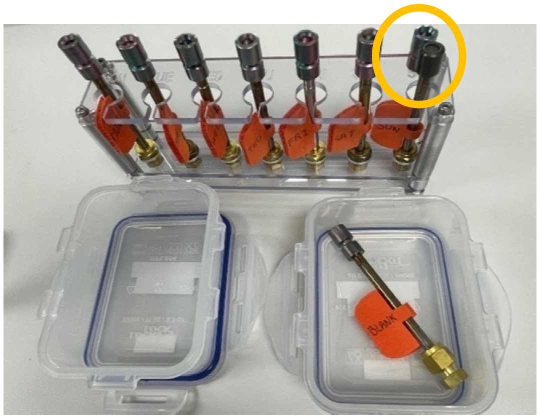

- At the end of the week, put back the large silver cap on the Sunday tube and place all the capped tubes in the relevant box (labelled long-term samples) with the small mesh cap. Put the boxes, rack and gloves in the provided package.

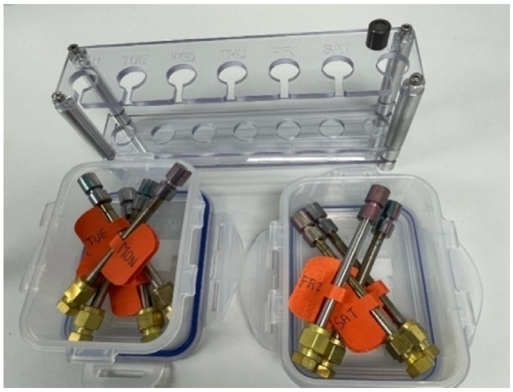

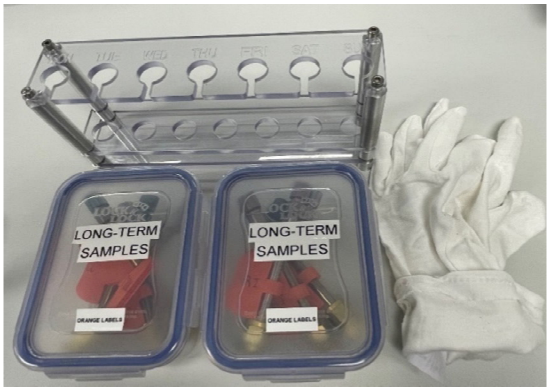

Supplement: Supplementary file 1 [file ijerph-22-01635-s001.zip › Supplementary Files S4.pdf]
