# Supplementary material for: INdoor Home Air Level Exploration (INHALE) Study: Protocol to Monitor Indoor Pollution in British Dwellings
Source: Int J Environ Res Public Health. 2025 Oct 27;22(11):1635. doi: 10.3390/ijerph22111635 (PMC12653005; doi:10.3390/ijerph22111635)
Supplement: Supplementary file 1 [file ijerph-22-01635-s001.zip › Supplementary Files S5.pdf]

Mould sampling protocol

- On Monday, place the sampler in your living room, at a height of approximately 1-1.5m.
- Take off the box lid, as shown in the picture below.

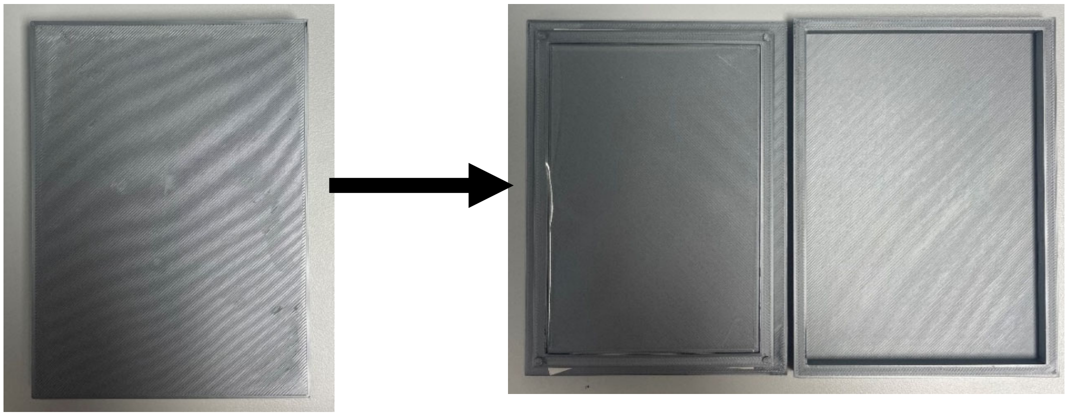

- Leave it open for a whole week.
- At the end of the week, put the lid back on and place it in the package.
