# Supplementary material for: INdoor Home Air Level Exploration (INHALE) Study: Protocol to Monitor Indoor Pollution in British Dwellings
Source: Int J Environ Res Public Health. 2025 Oct 27;22(11):1635. doi: 10.3390/ijerph22111635 (PMC12653005; doi:10.3390/ijerph22111635)
Supplement: Supplementary file 1 [file ijerph-22-01635-s001.zip › Supplementary Files S6.pdf]

Dust sampling protocol

We ask you to collect dust by vacuuming a small section of the floor in your living room and bedroom. Please do this on any day, but before you clean your floor, to ensure you collect some dust. To do this sampling, you need a compatible vacuum cleaner that fits our adapter.

- Take the sampler from the plastic bag.

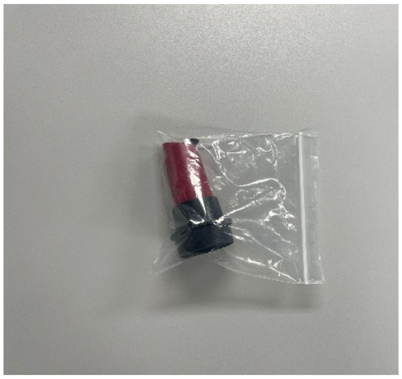
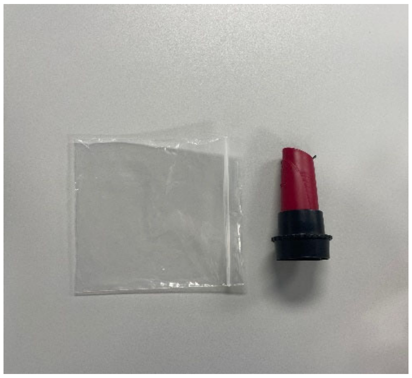

- Take off the lid of the sampler as shown in the picture below by pulling the tab.

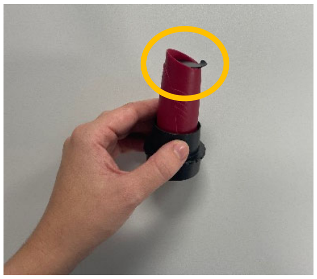
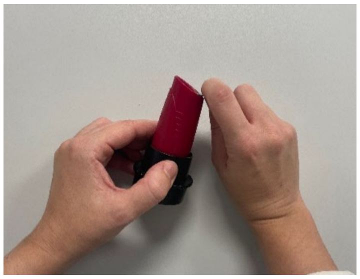
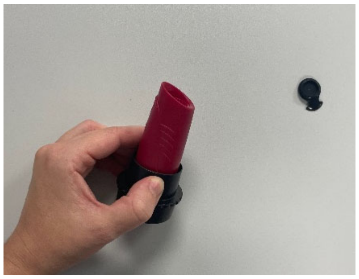

- Remove your vacuum accessory from the end of the wand.

- Put the adapter in its place, as in the picture below. If the adapter is too big, use the tape provided to secure it.

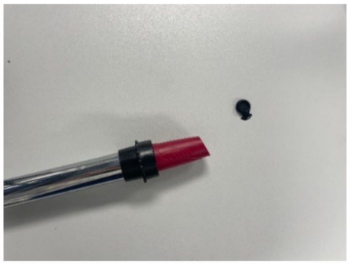
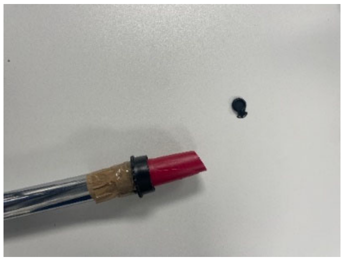
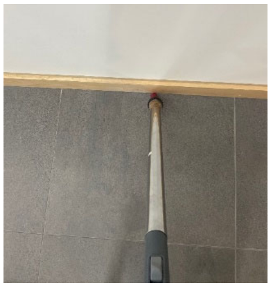

- Turn ON your vacuum cleaner, and vacuum each corner of the room for 30 seconds.

- Take the sampler off the wand, be careful not to let the dust fall out.

- Put the lid back on the sampler. Push with your finger to ensure that it is well-fixed.

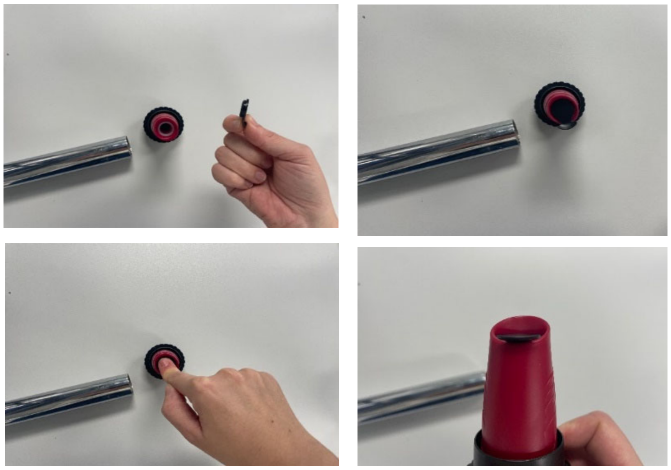

- Put the sampler back in its plastic bag and repeat the same protocol for the second room.
