# Supplementary material for: INdoor Home Air Level Exploration (INHALE) Study: Protocol to Monitor Indoor Pollution in British Dwellings
Source: Int J Environ Res Public Health. 2025 Oct 27;22(11):1635. doi: 10.3390/ijerph22111635 (PMC12653005; doi:10.3390/ijerph22111635)
Supplement: Supplementary file 1 [file ijerph-22-01635-s001.zip › Supplementary Files S8.pdf]

Particulate sampling protocol

You should have a black rectangular sampler, as shown in the picture below, and a cable.

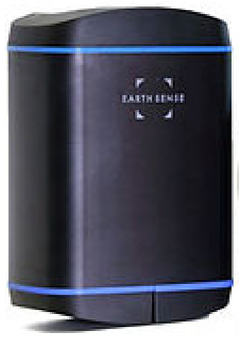

- Place the sampler in your living room, at a height of approximately. 1-1.5m.
- Plug in the charger.
- The sampler should be pre-set up by the investigator and will start on its own.
- At the end of the week, unplug the sampler, disconnect the charger and put it with the sampler in the provided package.
