# Supplementary material for: INdoor Home Air Level Exploration (INHALE) Study: Protocol to Monitor Indoor Pollution in British Dwellings
Source: Int J Environ Res Public Health. 2025 Oct 27;22(11):1635. doi: 10.3390/ijerph22111635 (PMC12653005; doi:10.3390/ijerph22111635)
Supplement: Supplementary file 1 [file ijerph-22-01635-s001.zip › Supplementary Files S9.pdf]

Other pollutants sampling protocol

- You should have a white sampler, as shown in the picture below, and a cable.

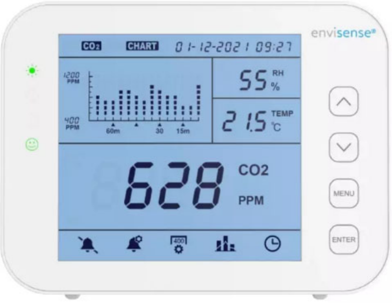

- Place the sampler in your living room, at a height of approximately. 1-1.5m.

- Plug the charger.

- The sampler should turn on, and a 30-second countdown will begin.

- The date and time should be set up in advance, but please double-check if it is correct.

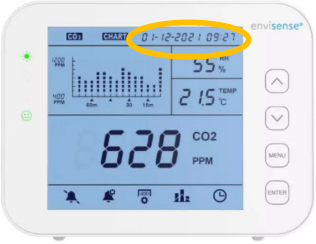

- If this is not the case, please press “MENU”. Press until the clock in the bottom right flashes (as shown in the picture), then push “ENTER”. The day should flash, please change it with the up and down arrow, then push “ENTER” and do the same for the month, year, hour and minute. When finished, please press “ENTER”.

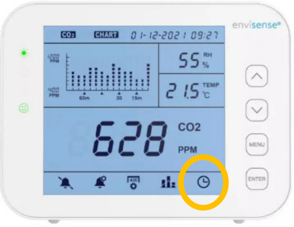

- At the end of the week, unplug the sampler, disconnect the charger and put it with the sampler in the provided package.
